# Role of Palliative Care in the Supportive Management of AL Amyloidosis—A Review

**DOI:** 10.3390/jcm13071991

**Published:** 2024-03-29

**Authors:** Muhammad Hamza Habib, Yun Kyoung Ryu Tiger, Danai Dima, Mathias Schlögl, Alexandra McDonald, Sandra Mazzoni, Jack Khouri, Louis Williams, Faiz Anwer, Shahzad Raza

**Affiliations:** 1 Department of Palliative Care, Rutgers Robert Wood Johnson School of Medicine, New Brunswick, NJ 08901, USA; 2 Division of Blood Disorders, Rutgers Cancer Institute of New Jersey, New Brunswick, NJ 08901, USA; 3 Department of Hematology & Oncology, Taussig Cancer Institute, Cleveland Clinic, Cleveland, OH 44195, USA; dimad@ccf.org (D.D.); mcdonaa6@ccf.org (A.M.); mazzons@ccf.org (S.M.); khourij@ccf.org (J.K.); willial47@ccf.org (L.W.); anwerf@ccf.org (F.A.); razas@ccf.org (S.R.); 4 Department of Geriatric Medicine, Clinic Barmelweid, 5017 Barmelweid, Switzerland; mathias.schloegl@barmelweid.ch

**Keywords:** AL amyloidosis, cardiomyopathy, mood disorders, nephrotic syndrome, neuropathy, palliation

## Abstract

Light chain amyloidosis is a plasma–cell disorder with a poor prognosis. It is a progressive condition, causing worsening pain, disability, and life-limiting complications involving multiple organ systems. The medical regimen can be complex, including chemotherapy or immunotherapy for the disease itself, as well as treatment for pain, gastrointestinal and cardiorespiratory symptoms, and various secondary symptoms. Patients and their families must have a realistic awareness of the illness and of the goals and limitations of treatments in making informed decisions about medical therapy, supportive management, and end-of-life planning. Palliative care services can thus improve patients’ quality of life and may even reduce overall treatment costs. Light chain (AL) amyloidosis is a clonal plasma cell disorder characterized by the excessive secretion of light chains by an indolent plasma cell clone that gradually accumulates in vital organs as amyloid fibrils and leads to end-organ damage. With progressive disease, most patients develop diverse clinical symptoms and complications that negatively impact quality of life and increase mortality. Complications include cardiac problems including heart failure, hypotension, pleural effusions, renal involvement including nephrotic syndrome with peripheral edema, gastrointestinal symptoms leading to anorexia and cachexia, complex pain syndromes, and mood disorders. The prognosis of patients with advanced AL amyloidosis is dismal. With such a complex presentation, and high morbidity and mortality rates, there is a critical need for the establishment of a palliative care program in clinical management. This paper provides an evidence-based overview of the integration of palliative care in the clinical management of AL amyloidosis as a means of reducing ER visits, rehospitalizations, and in-hospital mortality. We also discuss potential future collaborative directions in various aspects of clinical care related to AL amyloidosis.

## 1. Introduction

The term “palliative” comes from the Latin word *pallium*, which means ‘to cloak’ or ‘to cover’ the symptoms [1]. Palliative care initially branched off from the more established hospice care ideology in the early 1970s and has become an essential service; a 2019 survey showed that 72% of hospitals in the US have a specialized palliative care team on staff [2]. Its growth has been similar in ambulatory settings, where a 2018 report showed that more than 95% of NCI Cancer centers have a dedicated outpatient palliative care program [3,4]. The World Health Organization (WHO) defines palliative care as ‘an approach that improves the quality of life (QOL) of patients and their families who are facing problems associated with life-threatening illness. It prevents and relieves suffering through the early identification, correct assessment, and treatment of pain and other problems, whether physical, psychosocial, or spiritual’ [5]. The American Society of Clinical Oncology supports palliative care as an integral component of management [6].

AL amyloidosis is a clonal plasma cell disorder arising from an indolent plasma cell clone secreting excessive protein light chains that accumulate systemically as amyloid fibrils, leading to end-organ damage [7]. It is estimated that about 4000 people in the US annually develop AL amyloidosis, often presenting with various advanced cardiac, renal, and central nervous system complications [8]. With progressive disease, most patients develop complications that detract from QOL and increase morbidity and overall disease mortality. A recent study showed that the ratio of survivors among all AL amyloidosis patients is approximately 43% at 5 years and 27% at 10 years [9]. In patients with stage IIIb disease (modified Mayo 2004 and Boston University staging systems), the prognosis is especially poor, with high rates of early death and short overall survival time [10].

Palliative care plays a vital role in the management of AL amyloidosis [11]. This paper provides an evidence-based review of this role and discusses the benefits of palliative care integration in the clinical management of amyloidosis. It also presents an overview of symptomatic management, goals-of-care discussions, and patient and caregiver support provided by palliative care services, and offers some perspectives on future collaboration.

## 2. Structure of the Palliative Care Team

Palliative care services are traditionally interdisciplinary teams comprised of physicians, nurse practitioners, physician assistants, social workers, and chaplains [12]. Extended team members may include physical therapists, occupational therapists, dietitians, and speech pathologists. Such a multifaceted team-based approach helps with covering several aspects of clinical care, including symptom management, patient and family support, and goals of care discussions in the advanced stage of illness. This approach also helps patients and caregivers in navigating the complicated healthcare system, assists them with coping skills, and prepares them for the next steps as patients move from the advanced to terminal disease stages [12].

The interdisciplinary approach also promotes communication between patients and their healthcare providers, and between various individual healthcare providers and teams [13]. Additionally, palliative care services support the primary and consultant services through ethical and moral issues associated with terminal patient care when management beyond the usual norms of medical practice becomes necessary [14].

## 3. Management of Symptoms and Complications in AL Amyloidosis

As discussed in the following sections, one main clinical challenge facing the interdisciplinary team is the management of diverse symptoms and complications, including gastrointestinal, cardiorespiratory, and renal problems, pain, and mood disturbances. These conditions, reflecting amyloid deposition in diverse organs and structures, cause distressing symptoms including anorexia, weight loss, cachexia, weakness, and dyspnea. Table 1 summarizes management guidelines.

### 3.1. Cardiorespiratory Symptoms

Cardiac amyloidosis can present with symptoms of heart failure, hypotension, and arrhythmias, and may have a severe impact on survival. Orthostatic hypotension in AL amyloidosis may result from amyloid deposition in the autonomic nerves [15]. Dyspnea may result from cardiac failure, amyloid-based lung parenchymal disease, or both in advanced multisystem disease [16]. For patients with heart failure, salt restriction, oxygen supplementation, and gentle physical therapy may be helpful in improving symptoms and functionality [16,17]. However, most patients require loop diuretics (e.g., furosemide) or aldosterone antagonists (e.g., spironolactone) to manage fluid overload. A peripheral vasoconstrictor such as midodrine can be used if needed to maintain blood pressure during diuresis [15]. Pyridostigmine (a reversible inhibitor of acetylcholinesterase) or droxidopa (norepinephrine prodrug) may be useful in managing autonomic neuropathy-associated hypotension [18,19]. Beta-blockers should be used with caution and may worsen outcomes. Renin-angiotensin system inhibitors are poorly tolerated due to orthostatic hypotension [20].

Heart failure is a serious complication of cardiac amyloidosis, but orthotopic heart transplant is rarely feasible; if performed, it must precede chemotherapy, which is poorly tolerated in the presence of cardiac amyloidosis, and then must be followed by chemotherapy to reduce the risk of relapse of cardiac involvement. In patients who are not candidates for transplant, chemotherapy to eradicate malignant plasma cells must be approached with careful dose titration for the sake of tolerability.

Malignant arrhythmias can be challenging in patients with AL amyloidosis, mainly because of the risk of severe hypotension with beta-blockers and non-dihydropyridine calcium channel blockers. Amiodarone is a useful alternative and is generally well tolerated [15].

Patients with lung parenchymal disease may gain improved comfort with steroids and pulmonary physical therapy [21]. In advanced cases, opioids may be helpful in reducing dyspnea, but there may be a need for a gradual increase in dosage as the goal of care moves towards maximizing comfort [22].

Pleural effusion, typically presenting with dyspnea, is a rare complication of patients with systemic amyloidosis, due either to heart failure or as a direct result of amyloid infiltration of the parietal pleural surface [23]. This complication is seldom responsive to treatment and often associated with poor prognosis. Diuretics may be helpful in pleural effusion secondary to heart failure but not in pleural amyloidosis. In diuretic–refractory pleural effusion, direct drainage of pleural fluid or pleurodesis can be considered [24].

### 3.2. Renal Involvement

Amyloid deposition in the kidney can cause a wide range of renal dysfunction, from asymptomatic proteinuria to nephrotic syndrome presenting with peripheral edema, ascites, and volume overload. Supportive care with compression stockings, leg elevation, and restriction of salt and fluid is recommended as initial management. If these measures are insufficient, treatment with a loop diuretic or aldosterone antagonist is warranted (although other renin–angiotensin–aldosterone inhibitors such as angiotensin-converting enzyme inhibitors and angiotensin II receptor blockers should generally be avoided due to the risk of hypotension). Albumin infusion can be considered if diuresis is limited by hypotension. Patients with nephrotic syndrome are at increased risk of venous thromboembolism, especially if albumin drops below 2.5 g/dL [18]. Prophylactic anticoagulation may be considered after careful assessment of the risk of bleeding diathesis in this population.

Hemodialysis may be required in patients who develop end-stage renal disease, but the prognosis in this situation is especially poor, as hemodialysis may itself contribute to the disease process in amyloidosis. Renal transplantation in patients with AL amyloidosis has yielded favorable results in patients who showed good hematological response to therapy [25]. 

### 3.3. Gastrointestinal (GI) Symptoms

Patients in advanced stages of AL amyloidosis often present with distressing symptoms of anorexia and cachexia [11]. Poor appetite, weight loss, muscle weakness, and decline in functional status are also common, most likely due to disease progression or toxicity from chemotherapy and other treatments [26]. Evaluation using a validated tool such as Patient Generated Subjective Global Assessment can help with early recognition of reversible causes of malnutrition [27], and nutritional counseling and dietitian referral can be helpful.

Some GI symptoms can be managed with supportive care. For example, dysmotility with nausea and vomiting may respond to dietary modification, small food volume, and low fiber and fat content. However, medical intervention is often necessary.

Poor appetite is typically treated with megestrol acetate. A 2013 Cochrane review found that treatment with megestrol resulted in weight gain in patients with anorexia-cachexia but incurred an increased risk of thromboembolic events and death [28]. Cannabidiol and tetrahydrocannabinol supplements may also be helpful in appetite stimulation, but clinical data have not demonstrated significant improvement in oral intake and functional status [29]. Mirtazapine has been used for cancer-related cachexia but clinical trials have not shown benefit in appetite stimulation [30]. Anabolic hormones and testosterone-based treatments have shown moderate benefit in improving lean body weight in cancer patients, although hepatic dysfunction may be a concern [31].

Nausea and vomiting are common in patients with AL amyloidosis. Ondansetron (serotonin 5-HT3 receptor blocker) may be helpful for chemotherapy-related nausea, and metoclopramide (prokinetic D2 blocker) may help with nausea from gastroparesis [32]. Other GI complications include diarrhea, steatorrhea, constipation, dysmotility, and abdominal pain due to amyloid deposition in the GI tract (most commonly in the small intestine) [33]. Constipation may also result from the use of opioids in pain management, and laxatives may be helpful in this situation [34]. For dysmotility, pro-kinetic agents such as erythromycin or metoclopramide may be helpful [35].

In severe cases, pseudo-obstructions can occur, which may require surgical intervention [35]. GI bleeding may be severe in patients with dialysis-related amyloidosis due to platelet dysfunction [34,35]. GI bleeding can also occur due to amyloid infiltration of small vessels, erosions, ulcerations, and defective hemostasis from factor X deficiency [36,37]. Parenteral nutrition can be considered in severe cases [35]. For patients with diarrhea and protein-losing enteropathy from amyloidosis, steroids and octreotide (octapeptide that mimics somatostatin) can be considered [38].

### 3.4. Neuropathic Pain

Peripheral neuropathy has been reported in up to one-third of patients with AL amyloidosis [39]. Patients may present with multiple pain-related issues secondary to neural deposition of amyloid fibrils, particularly in peripheral nerves [40]. Neuropathic pain typically presents with tingling, burning, and pins-and-needles sensations in the extremities. Pain is often associated with localized or larger areas of numbness [40]. These symptoms are often more prominent in a colder environment [41]. This combination of pain and numbness often leads to inactivity, functional decline, and subsequent risk of falls due to poor proprioception and muscle loss [42]. Patients may also experience restless legs syndrome at night. Pain may be rapidly progressive in patients with preexisting diabetic neuropathy, thus mandating attention to minimizing treatment-related neuropathy [43].

Pain regimens include anticonvulsants, selective serotonin-norepinephrine reuptake inhibitors (SNRIs), tricyclic antidepressants (TCAs), and topical anesthetics. Because neuropathic pain is more resistant to treatment than somatic pain, drug combinations are often used for maximum pain control [44].

The anticonvulsant gabapentin can reduce neuropathic pain by antagonistic binding to the alpha 2 delta subunits of presynaptic calcium channels, reducing the excitatory neurotransmitters that potentiate the perception of pain [45,46]. Efficacy in the management of neuropathic pain has been studied mainly in patients with diabetic neuropathy, who had reduced pain, improved sleep, and positive effects on mood and QOL [46]. A review of five randomized trials found gabapentin to be effective and well tolerated at dosages of 1800 to 3600 mg daily in adults with neuropathic pain [47]. Once-daily formulations can reduce pill burden and improve medication compliance [48]. Pregabalin is the active metabolite of gabapentin, with a similar mechanism of action [49]. In controlled clinical trials, pregabalin was superior to placebo in pain reduction and subsequent sleep improvement in patients with neuropathic pain [50], and was well tolerated in doses up to 600 mg/day [51]. Typical side effects with both agents include fatigue, sedation, pedal edema, nausea, and constipation. To avoid withdrawal symptoms with discontinuation, dosages of these agents should be down-titrated in a manner similar to initial up-titration [52].

The TCAs amitriptyline, nortriptyline, and desipramine can reduce neuropathic pain by inhibiting the reuptake of norepinephrine and serotonin, reducing the activity of nociceptive neurons [53]. These agents may be considered for the treatment of debilitating neuropathy in patients with non-cardiac amyloidosis. However, they are generally unsuitable for elderly patients due to such adverse effects as dry mouth, constipation, sedation, hypotension, urinary retention, altered mental status, impaired glycemic control, and QTc prolongation (a particular concern in patients with amyloidosis and comorbid cardiomyopathy) [54]. Another concern is the precipitation of serotonin syndrome in patients who are also taking other serotonergic medications [55]. Consequently, these agents should probably not be considered first-line therapy in this population. However, when used, initial low dosages can be titrated up as tolerated, and discontinuation should be by gradual dose reduction [56].

The SNRIs work by a mechanism similar to that of the TCAs. Duloxetine is used for neuropathic pain [57] and is currently recommended as first-line treatment for chemotherapy-induced peripheral neuropathy [58], reflecting efficacy and safety data from a 2009 Cochrane review [59] and a 2013 study in patients with chemotherapy-induced neuropathy [60]. Discontinuation from higher dosages should be by gradual tapering to prevent withdrawal symptoms [61]. Venlafaxine is available in extended-release oral formulation for once-daily dosing. SNRIs have the additional benefit of helping with mood disturbances [62]. Typical side effects of these agents include fatigue, jitteriness, sedation, and bruxism. Note that TCAs, SNRIs, and selective serotonin reuptake inhibitors can cause sexual dysfunction. Also, they raise the risk of serotonin syndrome if used concomitantly with other serotonergic medications [55].

Topical analgesic modalities for neuropathic pain include lidocaine and capsaicin-based formulations. Lidocaine blocks the sodium channels to prevent the generation of nerve impulses from peripheral nociceptors [63]. Although individual studies have reported clinical benefits, a 2014 Cochrane review found limited high-quality evidence to support the use of lidocaine patches in neuropathic pain [64]. Capsaicin works by desensitizing the TRPV1 receptor and possibly by depleting local substance P to reduce pain sensation and transmission of pain impulses [65]. Capsaicin patches are available without prescription in lower-concentration formulations and by prescription for higher concentrations [66]. A Cochrane review from 2012 found moderate-quality evidence supporting the use of high-concentration capsaicin for neuropathic pain [67]. Side effects of topic agents include local skin irritation, rash, dryness, numbness, and allergic reactions.

Although opioids are used primarily in cancer-related visceral and somatic pain, they can produce short- to intermediate-term benefits in the management of neuropathic pain [68,69]. Opioids’ action on the μ-receptors accounts for both therapeutic and adverse effects, which may be more serious in elderly patients [70,71,72]. Overdose may cause altered mental status, sedation, and respiratory depression, especially in patients without opioid tolerance [73]. Patients receiving opioids must be screened for misuse on a validated scale, and state databases monitored for controlled substance compliance. Discussion of risks, counseling on safe usage, and opioid agreements are recommended for safe clinical use, including co-prescription of naloxone if required by state law [74].

### 3.5. Visceral and Somatic Pain

Less common than neuropathic pain, advanced AL amyloidosis patients may present with visceral pain due to amyloid deposition in internal organs [75], somatic pain due to amyloid deposition in muscles and joints causing arthralgias or carpel tunnel syndrome, and myofascial pain due to disuse atrophy [76].

Visceral and somatic pain in advanced AL amyloidosis is often managed with opioids [77]. Interventional approaches include nerve blocks, ablation, and spinal cord stimulation [78]. Targeted treatments such as nitrates, diuretics, and beta-blockers may also be needed to assist with cardiac pain in advanced cardiomyopathies [16,17].

First-line therapy for somatic pain includes acetaminophen at low-to-moderate dosage while monitoring liver function. Nonsteroidal anti-inflammatory drugs (NSAIDs) may provide a greater analgesic effect but incur greater risk from concomitant cardiac or renal disease [79]. NSAIDs use can be considered in patients who have AL amyloidosis without significant kidney involvement. This should be considered on a case-by-case basis. Systemic or intraarticular corticosteroids may help with joint pain [78]. For myofascial pain from disuse atrophy and muscle wasting physical and occupational therapy can preserve safe ambulation and functional status while reducing caregiver burden [80,81]. Other options for myofascial pain include gabapentin, pregabalin, and duloxetine, as discussed above.

### 3.6. Mood Disturbances

Anxiety and depression may occur as a result of advancing disease or as a pre-existing condition that is worsened by clinical decline and impaired functionality. Counseling, support, early institution of specialized psychiatric services, and the use of selective serotonin reuptake inhibitors (SSRIs) may be helpful in managing these symptoms and improving overall QOL [11]. The SNRI duloxetine may help with both mood disturbances and neuropathic pain [54,62]. However, it should be noted that TCAs, SNRIs, and SSRIs have all been linked to sexual dysfunction.

### 3.7. Sexual Health Symptoms

Sexual dysfunction is a common but often overlooked symptom in patients dealing with advanced life-limiting illnesses. It may be secondary to medication side effects (for example, with standard antidepressants), overall functional debility, autonomic dysfunction, and anxiety and depression [11]. Optimizing the medication regimen and use of testosterone supplementation may be helpful in the management of such symptoms. Testosterone supplementation may also help with muscle gain and reduction in fatigue [31].

## 4. Integrating Palliative Care into the Management of AL Amyloidosis

In addition to the management of amyloidosis-related symptoms, palliative care services can facilitate communication within the interdisciplinary team and between the team and the patient and family. The oncologist in charge of the case calls in the palliative care team to work with the primary and consulting clinical teams in dealing with ethical issues associated with a progressive illness and its associated burdens on the patient and caregivers [13,14], advocating for a collaborative holistic approach directed towards QOL [82,83]. In general, the frequency and early onset of serious complications in AL amyloidosis warrant early involvement of the palliative care team. 

An important aspect of care is the discussion of goals, especially pertaining to end-of-life issues. In the initial phases of illnesses, the focus is on longevity, but disease progression calls for serious and open discussion with the patient and family about the overall state of illness and realistic prognostic expectations [82]. The values and goals of patients and their families must be addressed in discussing options for maximizing QOL [83]. Components of goals-of-care discussions in AL amyloidosis are described in Table 2.

Advanced care directives must anticipate future disease trajectory and its outcomes. This planning includes the completion of paperwork related to code status that covers cardiopulmonary resuscitation, use of artificial means of nutrition, use of various inotropic and chronotropic medications, and future diagnostic and therapeutic interventions based on benefit versus harm [84]. Additionally, healthcare proxies must be identified to make decisions if and when patients can no longer make their own healthcare decisions [84]. Per patient preference, options for medical aid in dying and physician-assisted suicide may be discussed as allowed by state laws [85]. Throughout this process, patients, family, and caregivers require counseling and support as they go through this extensive decision-making process.

Social workers and chaplain colleagues in clinical palliative care services evaluate, counsel, and support the patient and family through the advanced stages of illness, and guide them through the complexities of healthcare systems [86,87]. A physical therapist, occupational therapist, or speech pathologist on the team can assist with improving the patient’s functional status to promote independence (including help with swallowing and speech in very advanced stages of illness) and to reduce caregiver burden [88].

Palliative care services can be provided as outpatient services in person or via telemedicine services if the patient is unable to come in person to the appointments due to advanced illness and debility [89]. For patients in the terminal stage of illness, options for home or hospital-based hospice care are also discussed. Once the patient and family are ready and agreeable, referral to hospice care services is made for end-of-life care [83].

Throughout this process, the palliative care team also helps and supports the primary and consulting teams in coping with ethical and moral distress associated with progressive illness [13,14]. In AL amyloidosis, where multiple clinical teams are involved, palliative care services can help in maintaining focus on the overall impact of care on the patient, advocating for a collaborative holistic approach directed towards QOL [82,83]. 

## 5. Impact of Palliative Care on Healthcare Outcomes

There are ample data showing that palliative care can reduce emergency visits, rehospitalization, and in-hospital mortality, which are important healthcare metrics [90,91,92,93]. Quantitative assessment of the impact of integrated interdisciplinary palliative care services is vital but sometimes difficult to measure. Individual clinical trials may show significant positive benefits but systematic reviews and meta-analyses do not always corroborate those findings.

A study in 186 oncology patients concluded that early integrated palliative care resulted in improved QOL from initiation to end-of-life [94]. Similarly, a randomized clinical trial of patients with acute myeloid leukemia reported substantial improvements in QOL, psychological distress, and end-of-life care [95].

On the other hand, a meta-analysis of the impact of palliative care interventions in 12,731 patients showed only a trend toward improvements in patients’ QOL and symptom burden and no correlation with survival [96]. Similarly, a Cochrane review showed only low-quality evidence of benefit to health-related QOL, symptom burden, and patient satisfaction [97]. Another review demonstrated that true interdisciplinary palliative care interventions are associated with improved QOL and cost savings, but these positive outcomes may not apply to services labeled “palliative” but not conforming to standards of true interdisciplinary palliative care [98].

In summary, integrated palliative care services can help reduce the overall cost of clinical care, which is especially relevant in the US, where healthcare costs are very high and insurance coverage tends to shrink, causing financial distress to patients and families through advancing stages of illness [99,100,101]. Healthcare quality and cost benefits with the integration of palliative care in AL amyloidosis are listed in Table 3.

## 6. Future Directions

There are growing opportunities for collaboration and cooperation between palliative care services and clinical services involved in the care of patients with advanced AL amyloidosis. Furthermore, there is definite clinical benefit in early integration of palliative care in the interdisciplinary team, in terms of improving symptoms, reducing distress, and improving patients’ overall perceived QOL [102]. Also, it provides an opportunity to collaborate in settings of Phases 1 and 2 drug development, where patients’ symptoms can be better managed and research-based therapeutics can be continued for longer periods of time. It gives the opportunity for joint meetings on clinical care especially in patients in whom invasive cardiac or renal interventions are being considered. Finally, such collaborative clinical care models help foster clinical research and educational opportunities for all clinical services involved, to the benefit of patients [103]. A summary of the integration of palliative care in the management of patients with AL amyloidosis is presented in Table 4.

## 7. Conclusions

Palliative care is a rapidly growing field, and its role in AL amyloidosis management ranges from pain and symptoms management as the disease continues to support through treatment. Palliative care services as part of the interdisciplinary team can help with goals-of-care discussion at advanced stages of illness and provide support to patients and families as the disease progresses to the terminal stage. Finally, these services can help in reducing healthcare costs and fostering opportunities for collaboration in education and research in the management of complex multisystem diseases.

## Figures and Tables

**Table 1:** Management of serious complications in AL amyloidosis.

System	Clinical Problem	Management Options
**Cardiovascular**	Heart failure, volume overload	Dietary modification. Loop diuretics, aldosterone antagonist. Orthotopic heart transplantation in rare cases. Midodrine for hypotension in individual cases.
	Arrhythmias	Amiodarone is effective and well-tolerated. Non-dihydropyridine calcium channel blockers and beta-blockers may cause hypotension, but low-dose beta-blocker may be considered in individual cases.
**Pulmonary**	Pleural effusion	Diuretics if due to amyloid cardiomyopathy. If nonresponsive to diuretics, consider direct drainage or pleurodesis.
**Gastrointestinal**	Diarrhea Constipation Nausea, vomiting	Dietary modifications. Pro-kinetic agents. Anti-emetics.
	Protein-losing enteropathy Anorexia, cachexia, weight loss	Consider steroids or octreotide. Megestrol acetate, cannabidiol/tetrahydrocannabinol supplements, mirtazapine, anabolic hormones or testosterone. Parental nutrition in individual cases.
**Neurological**	Autonomic dysfunction	Midodrine, droxidopa, pyridostigmine for hypotension.
	Neuropathic pain	Anticonvulsants (gabapentin, pregabalin). TCAs, SNRIs, SSRIs. Topical anesthetics (lidocaine, capsaicin patches). Opioids in individual cases.
**Renal**	Nephrotic syndrome	Salt and fluid restriction, compression stockings. Loop diuretics, aldosterone antagonist. If no evidence of heart failure, consider renin-angiotensin system inhibitors. Consider VTE prophylaxis.
	End-stage renal failure	Hemodialysis, renal transplant.
**Endocrine**	Sexual dysfunction	Optimization of medication regimen. Testosterone supplementation.

**Table 2:** Goals of Care Discussions in Advanced AL Amyloidosis.

Components of Goals of Care Discussions
- Looking at the bigger picture and prognosticating based on clinical and functional trajectory. - Understanding patient and family ethical, cultural, moral, and spiritual values. - Having serious and open discussions with the patient and family about the overall state of illness and setting realistic prognostic expectations. - Completion of paperwork related to code status and healthcare proxies. - Discussing appropriate options for medical aid in dying and physician assisted suicide. - Counselling and supporting patients and their families through decisions regarding comfort-care options. - Coordinating with other clinical and non-clinical services to help patients and families navigate through complexities of the healthcare system. - Discussing options for home or hospital-based hospice care and making appropriate referrals. - Helping and supporting the primary and other consulting teams through various ethical and moral distress associated with progressive illness and associated suffering.

**Table 3:** Impact of Integrating Palliative Care in AL Amyloidosis.

Healthcare Quality and Cost Benefits
- Reduction in ER visits with good pain and symptom care. - Reduction in rehospitalizations and in-hospital mortality with focus on comfort care. - Reduction in cost of clinical care with greater focus on benefit-versus risk-and-cost model for expensive interventions in terminal stages. - Lesser financial distress to patients and families through advancing stages of illness.

**Table 4:** Future Directions for Palliative Care in Management of AL Amyloidosis.

Future Directions for Palliative Care in AL Amyloidosis
- Integrating earlier palliative care to reduce symptoms and improve overall QOL. - Growing clinical collaboration in Phase 1 and 2 drug development to improve opportunity for longer enrollments through better management of pain and other symptoms. - Regular joint meetings on clinical care involving cardiac/renal transplantation, and initiation of ventricular assistance devices and renal replacement therapy. - Collaborative outcomes-based clinical research in patients with AL amyloidosis. - Growing educational and clinical training opportunities with mandatory rotations in palliative care for transplant and surgical cardiology/nephrology services.

## Data Availability

All the data reported in this article are cited under references.
